# Supplementary material for: A Molecular Stratification of Chilean Gastric Cancer Patients with Potential Clinical Applicability
Source: Cancers (Basel). 2020 Jul 10;12(7):1863. doi: 10.3390/cancers12071863 (PMC7408697; doi:10.3390/cancers12071863)
Supplement: Supplementary file 1 [file cancers-12-01863-s001.pdf]

Supplementary Materials

A Molecular Stratification of Chilean Gastric Cancer Patients with Potential Clinical Applicability

Mauricio P. Pinto, Miguel Córdova-Delgado, Ignacio N. Retamal, Matías Muñoz-Medel, M. Loreto Bravo, Doris Durán, Francisco Villanueva, César Sanchez, Francisco Acevedo, Sebastián Mondaca, Érica Koch, Carolina Ibañez, Héctor Galindo, Jorge Madrid, Bruno Nervi, José Peña, Javiera Torres and Marcelo Garrido

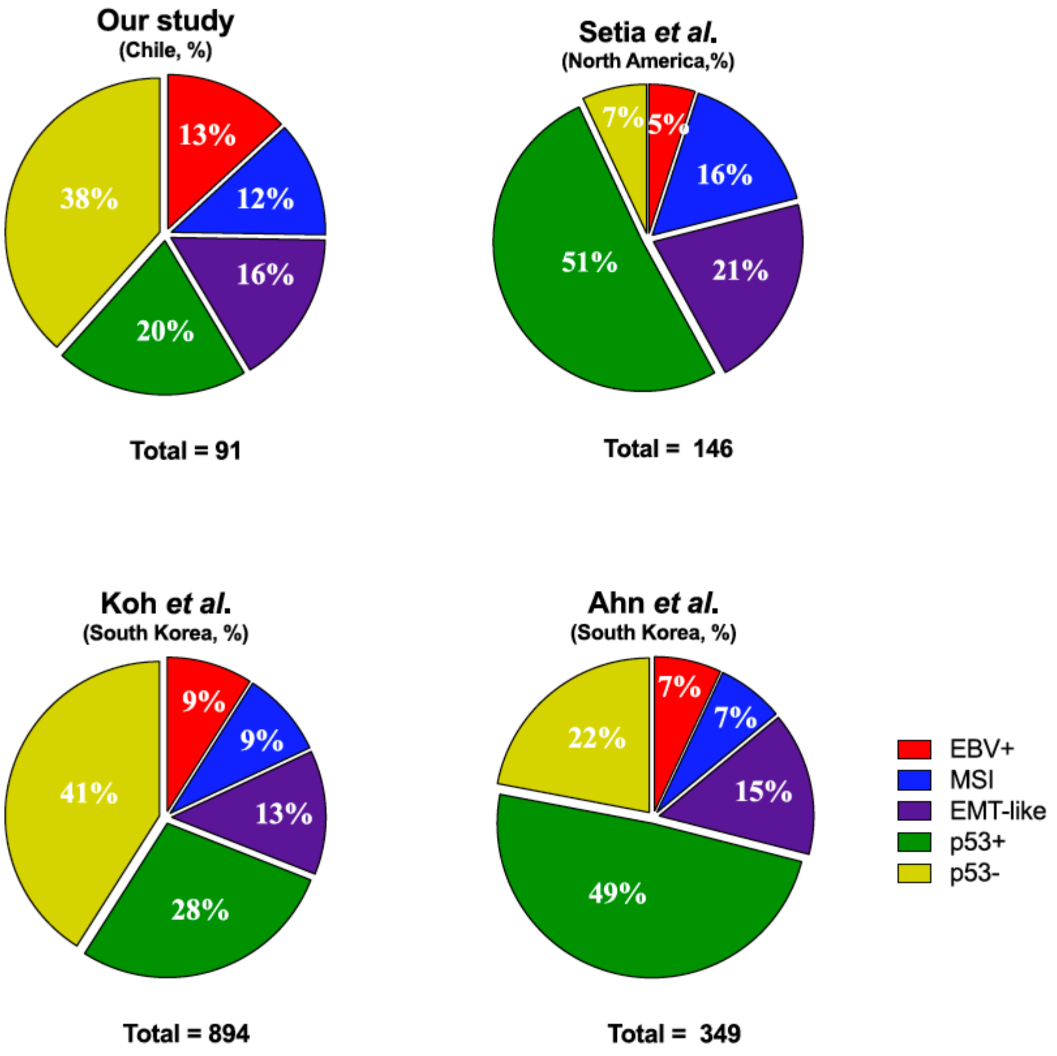

Figure S1. Distribution of gastric cancer molecular subtypes among Chilean, North American or Asian studies.

≥10% and 2+ or 3+

ID	P53_NGS		P53_IHC	
	Kind of mutation	AA change	% cel	Intensity (1-3)
GCTF00003T	**nonsense**	p.Arg342*	0	0
GCTF00005T	**nonsense**	p.Arg306*	0	0
GCTF00016T	**nonsense**	p.Arg196*	0	0
GCTF00020T	**frameshiftInsertion**	p.Ser240fs	0	0
GCTF00044T	**nonsense**	p.Arg213*	0	0
GCTF00053T	missense	p.Ser183Leu	10	1
GCTF00068T	**nonsense**	p.Arg213*	0	0
GCTF00072T	missense	p.Tyr220His	20	1
GCTF00082T	missense	p.Gly154Ser	0	0
GCTF00096T	**frameshiftInsertion**	p.Tyr327fs	10	1
GCTF00108T	**frameshiftInsertion**	p.Pro223fs	0	0
GCTF00119	**frameshiftInsertion**	p.Arg158fs	0	0

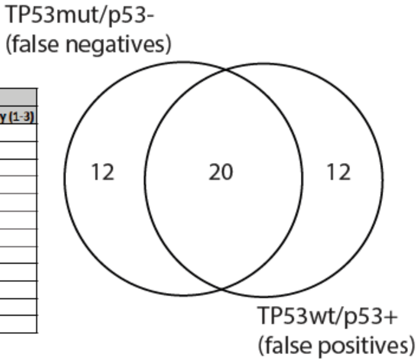

ID	P53_IHC		P53_NGS
	% cel	Intensity (1-3)	
GCTF00027	40	2	Wild type
GCTF00031	10	2	Wild type
GCTF00038	10	3	Wild type
GCTF00055	10	3	Wild type
GCTF00090	90	3	Wild type
GCTF00103	10	2	Wild type
GCTF00111	10	3	Wild type
GCTF00114	60	2	Wild type
GCTF00115	10	3	Wild type
GCTF00116	10	2	Wild type
GCTF00118	10	2	Wild type
GCTF00125	10	2	Wild type

≥20% and 2+ or 3+

ID	P53_NGS		P53_IHC	
	Kind of mutation	AA change	% cel	Intensity (1-3)
GCTF00003T	**nonsense**	p.Arg342*	0	0
GCTF00005T	**nonsense**	p.Arg306*	0	0
GCTF00016T	**nonsense**	p.Arg196*	0	0
GCTF00020T	frameshiftInsertion	p.Ser240fs	0	0
GCTF00044T	**nonsense**	p.Arg213*	0	0
GCTF00053T	missense	p.Ser183Leu	10	1
GCTF00068T	**nonsense**	p.Arg213*	0	0
GCTF00072T	missense	p.Tyr220His	20	1
GCTF00082T	missense	p.Gly154Ser	0	0
GCTF00096T	**frameshiftInsertion**	p.Tyr327fs	10	1
GCTF00108T	**frameshiftInsertion**	p.Pro223fs	0	0
GCTF00119	**frameshiftInsertion**	p.Arg158fs	0	0

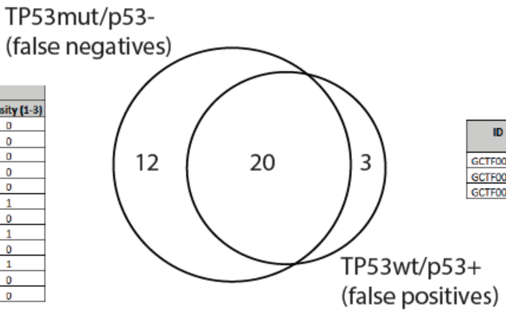

ID	P53_IHC		P53_NGS
	% cel	Intensity (1-3)	
GCTF00027	40	2	Wild type
GCTF00090	90	3	Wild type
GCTF00114	60	2	Wild type

≥20% and 2+ or 3+ or 0% and 0+ (complete loss) like Setia *et al.*

ID	P53_NGS		P53_IHC	
	Kind of mutation	AA change	% cel	Intensity (1-3)
GCTF00053T	missense	p.Ser183Leu	10	1
GCTF00072T	missense	p.Tyr220His	20	1
GCTF00096T	**frameshiftInsertion**	p.Tyr327fs	10	1

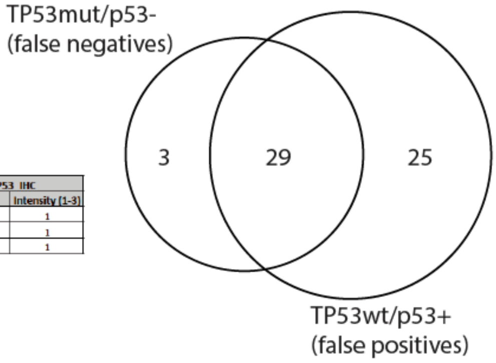

ID	P53_IHC		P53_NGS
	% cel	Intensity (1-3)	
GCTF00001	0	0	Wild type
GCTF00006	0	0	Wild type
GCTF00007	0	0	Wild type
GCTF00011	0	0	Wild type
GCTF00019	0	0	Wild type
GCTF00023	0	0	Wild type
GCTF00024	0	0	Wild type
GCTF00027	40	2	Wild type
GCTF00028	0	0	Wild type
GCTF00033	0	0	Wild type
GCTF00040	0	0	Wild type
GCTF00042	0	0	Wild type
GCTF00043	0	0	Wild type
GCTF00045	0	0	Wild type
GCTF00046	0	0	Wild type
GCTF00047	0	0	Wild type
GCTF00076	0	0	Wild type
GCTF00089	0	0	Wild type
GCTF00090	90	3	Wild type
GCTF00106	0	0	Wild type
GCTF00112	0	0	Wild type
GCTF00114	60	2	Wild type
GCTF00121	0	0	Wild type
GCTF00127	0	0	Wild type
GCTF00130	0	0	Wild type

Figure S2. Comparison of p53 false positive/negatives using different criteria.

Table S1. clinico-pathological characteristics in *TP53* Wild type or mutant patients.

Characteristic	*TP53*-WT	*TP53*-Mut
Median OS: months (range)	40 (26–52)	23 (13–53)
Males; *n* (%)	12 (57)	13 (65)
Age (median, IQT)	64 (58–77)	63 (54–67)
Stage; *n* (%)		
I/II	4 (19)	8 (40)
III	14 (67)	9 (45)
IV	3 (14)	3 (20)
Primary tumor; *n* (%)		
Proximal	3 (15)	7 (39)
Medial	9 (45)	4 (22)
Distal	7 (35)	6 (33)
Multiple	1 (5)	1 (6)
IHC status; *n* (%)		
HER2+	1 (5)	8 (40)
PDL1+	3 (14)	3 (15)
P53+	8 (38)	14 (70)
Signet ring cell; *n* (%)		
No	12 (57)	14 (70)
<50%	6 (29)	5 (25)
≥50%	3 (14)	1 (5)
Lauren histotype; *n* (%)		
Diffuse	9 (50)	4 (22)
Intestinal	7 (39)	11 (61)
Mixed	2 (11)	3 (17)

IQT interquartile range, *IHC* immunohistochemistry.

Table S2. Response assessment to first line peri-operative/palliative chemotherapy by RECIST v1.1 (*n*=32).

RECIST v1.1	MMR-D *n* = 2	EBV+ *n* = 5	*TP53*-WT *n* = 8	*TP53*-Mut *n* = 8	EMT-like *n* = 9	Total *n* = 32	*p*-Value
**CR/PR**	0	4 (80)	3 (37.5)	5 (62.5)	6 (66.7)	20 (56)	0.31
SD/PD	2 (100)	1 (20)	5 (62.5)	3 (37.5)	3 (33.3)	16 (44)	

CR complete response, *PR* partial response, *SD* stable disease, *PD* progressive disease.

© 2020 by the authors. Licensee MDPI, Basel, Switzerland. This article is an open access article distributed under the terms and conditions of the Creative Commons Attribution (CC BY) license (http://creativecommons.org/licenses/by/4.0/).
